# Accelerated Development With Increased Bone Mass and Skeletal Response to Loading Suggest Receptor Activity Modifying Protein-3 as a Bone Anabolic Target

**DOI:** 10.3389/fendo.2021.807882

**Published:** 2022-01-12

**Authors:** Suruchi Pacharne, Matthew Livesey, Mahita Kadmiel, Ning Wang, Kathleen M. Caron, Gareth O. Richards, Tim M. Skerry

**Affiliations:** ^1^ Department of Oncology and Metabolism, School of Medicine, University of Sheffield, Sheffield, United Kingdom; ^2^ Department of Cell Biology and Physiology, University of North Carolina at Chapel Hill, Chapel Hill, NC, United States

**Keywords:** receptor activity modifying protein, bone, endocrinology, osteoporosis, ageing

## Abstract

Knockout technologies provide insights into physiological roles of genes. Studies initiated into endocrinology of heteromeric G protein-coupled receptors included deletion of receptor activity modifying protein-3, an accessory protein that alters ligand selectivity of calcitonin and calcitonin-like receptors. Initially, deletion of *Ramp3^-/-^* appeared phenotypically silent, but it has emerged that mice have a high bone mass phenotype, and more subtle alterations to angiogenesis, amylin homeostasis, and a small proportion of the effects of adrenomedullin on cardiovascular and lymphatic systems. Here we explore in detail, effects of *Ramp3^-/-^* deletion on skeletal growth/development, bone mass and response of bone to mechanical loading mimicking exercise. Mouse pups lacking RAMP3 are healthy and viable, having accelerated development of the skeleton as assessed by degree of mineralisation of specific bones, and by microCT measurements. Specifically, we observed that neonates and young mice have increased bone volume and mineralisation in hindlimbs and vertebrae and increased thickness of bone trabeculae. These changes are associated with increased osteoblast numbers and bone apposition rate in *Ramp3^-/-^* mice, and increased cell proliferation in epiphyseal growth plates. Effects persist for some weeks after birth, but differences in gross bone mass between RAMP3 and WT mice lose significance in older animals although architectural differences persist. Responses of bones of 17-week old mice to mechanical loading that mimics effects of vigorous exercise is increased significantly in *Ramp3^-/-^* mice by 30% compared with WT control mice. Studies on cultured osteoblasts from *Ramp3^-/-^* mice indicate interactions between mRNA expression of RAMPs1 and 3, but not RAMP2 and 3. Our preliminary data shows that *Ramp3^-/-^* osteoblasts had increased expression β-catenin, a component of the canonical Wnt signalling pathway known to regulate skeletal homeostasis and mechanosensitivity. Given interactions of RAMPs with both calcitonin and calcitonin-like receptors to alter ligand selectivity, and with other GPCRs to change trafficking or ligand bias, it is not clear whether the bone phenotype of *Ramp3^-/-^* mice is due to alterations in signalling mediated by one or more GPCRS. However, as antagonists of RAMP-interacting receptors are growing in availability, there appears the likelihood that manipulation of the RAMP3 signalling system could provide anabolic effects therapeutically.

## Introduction

Increased lifespan poses new challenges for the population. The larger numbers of older old people in societies have the potential to increase those needing assistance with living. One major cause of loss of ability to live independently is related to musculoskeletal diseases where bone and joint problems necessitate assisted living. Bone fractures due to osteoporosis lead to hospitalisation (with its own morbidity) but also a failure to return to previous physical ability to manage independent living. Osteoporosis is a result of an imbalance between bone resorption and bone formation. Therapeutic strategies have traditionally been based on the success of antiresorptive agents such as bisphosphonates (1–3), selective estrogen receptor modulators (SERMs) and anti-RANKL monoclonal antibodies (4–6), but there is strong interest in ways to increase skeletal mass in patients who have already lost bone. Parathyroid hormone and related peptides (PTH 1-34 -teriparatide and a parathyroid hormone-related protein PTHrP 1-34 analogue abaloparatide) (7–9) were among the first anabolic treatments approved for osteoporosis and have proved successful in reversing bone loss in many, but not all those suffering from osteoporosis (8, 10, 11). However, due to a suggested risk of developing osteosarcoma with teriparatide and abaloparatide, a cumulative use of these drugs for more than 2 years is not recommended (12–15). Despite the long development times and costs for osteoporosis drugs, there is still scope for development of novel basic scientific discoveries relating to bone homeostasis that may be translated as targets for drug discovery, particularly if they offer anabolic therapeutic opportunities.

Here we describe the phenotype of mice lacking the gene for RAMP3, which have elevated bone mass and increased responses to mechanical loading compared with WT controls. This research arose from considerations of the roles of the calcitonin family of peptides – calcitonin (CT), calcitonin gene-related peptide (CGRP), Adrenomedullin (ADM) and Amylin (AMY). Calcitonin (CT) is a well-established physiological regulator of the skeleton, and receptors are expressed on both osteoclasts and osteoblasts (16–18). CGRP, ADM, AMY and intermedin (IMD/ADM2) also regulate skeletal homeostasis. CGRP and AMY have been shown to inhibit osteoclast activity and bone resorption *in vitro* (19–21). Several studies have also demonstrated that AMY, ADM and CGRP induce osteoblast proliferation and promote bone formation (18, 22–25). IMD/AM2 has been reported to inhibit bone resorption in mouse calvarial bone and reduce osteoclast numbers and pit formations in bone marrow macrophage cultures that were stimulated with M-CSF and RANKL (26). Recently, ADM2 has been reported to improve bone regeneration in type1 diabetic rats (27). The receptors for the family are unusual because they comprise not a single G protein-coupled receptor (GPCR) but GPCRs associated with accessory proteins known as a receptor activity modifying proteins (RAMPs), where the RAMPs provides the ligand selectivity for the heteromeric receptor complex. The mechanisms behind these effects are still not fully understood, but detailed research on the structure and function of the complexes is emerging with consequent impact (28, 29).

Generally, the ligand properties of the CT peptides on their receptors (calcitonin receptor - CTR and calcitonin like-receptor - CLR), rely on their interactions with one of the three human receptor activity modifying proteins (RAMPs) that affect their trafficking to the cell surface (30), ligand binding and intracellular second messenger activation pathways. The CTR is an exception where RAMP interactions are not needed for cell surface translocation, but it becomes one of 3 receptors for amylin when interacting with the RAMPs (31–33). The CLR has no known native ligand and does not traffic to the cell surface alone, so requires an association with the RAMPs for cell surface expression and function. CLR/RAMP1 heterodimers form receptors for calcitonin gene-related peptide (CGRP), whereas CLR and RAMPs 2 and 3 form pharmacologically and functionally distinct adrenomedullin receptors (32–35). Studies performed to investigate peptide-ligand interactions in the absence and presence of each RAMPs, suggest a common mode of peptide binding in the CLR-RAMP complexes and an allosteric role of RAMPs driving the peptide selectivity *via* increasing conformational flexibility in CTR-RAMP complexes (35).

Genetic mouse models for individual *Ramp* genes have revealed that perturbation from normal expression of individual *Ramp* genes results in distinct phenotypes (30, 32, 33). In 2007, there were first reports of the *Ramp1^-/-^* (36) and *Ramp2^-/-^*, *Ramp2^+/-^* and the *Ramp3^-/-^* mouse models (37) which improved understanding of physiological relevance of the *Ramp* genes. *Ramp1^-/-^* mice exhibit a phenotype characterised by hypertension and increased serum pro-inflammatory cytokine levels (TNF-α, IFN-γ, IL-12, and IL-6) with increased serum CGRP levels upon lipopolysaccharide (LPS) administration and indications of disturbed metabolism (36). *Ramp2^-/-^* mice have an embryonically lethal phenotype and die mid-gestation due to hydrops fetalis, phenocopying AM and CLR null mice (38–40). Even a single copy of the *Ramp3* gene does not rescue these effects as a haploid insufficiency phenotype includes reduced litter size, increased serum calcium and prolactin levels, disorders of mammary gland development and pituitary gland size (37, 41). Notably, *Ramp2^+/-^* neonates also exhibit a skeletal phenotype characterised by reduced mineralization of the epiphyseal plates of the vertebrae suggestive of delayed development of the lumbar vertebrae (42). Interestingly, and in direct contrast, *Ramp3^-/-^* mice are healthy and viable with a phenotype similar to the WT mice except that these mice failed to exhibit normal physiological age-related weight gain (37) as they age. In this study, we analysed the skeletal phenotype of *Ramp3^-/-^* mice, using microcomputed tomography and dynamic histomorphometry to assess postnatal bone growth/development and responses to mechanical loading in adulthood, and *in vitro* PCR analysis of osteoblasts from *Ramp3^-/-^* and WT mice to determine RAMP and β-catenin mRNA expression.

## Methods

### Experimental Animals

Sex-matched WT 129/SvEv and *Ramp3^-/-^* mice were bred from on the 129/SvEv null mice created previously (37). Skeletal phenotype was studied at post-natal day 5 (PND5), and at 4 and 8 weeks of age. 17-week-old animals were used for mechanical loading experiments. Whole mount skeletal staining was performed for the PND5 age group, micro computed tomography (μCT) analysis was performed for all age groups, and histology and dynamic histomorphometry were performed for the eight-week age group. All experimental animals were housed in individually ventilated cages (IVC) under 12-hour light/dark cycle with ab libitum access to water and approved regular chow diet in pathogen free environment specific to the animal house facility at University of Sheffield. Each IVC housed a maximum 5 mice at a time. All procedures were approved by a local ethical committee, and according to the UK Home Office regulations under the authority of associated project and personal licences for animal experiments.

Neonatal mice were euthanised by anaesthetic overdose of sodium pentobarbitone solution – (0.2mg/g body weight - JM Loveridge Ltd, UK). Adult mice were euthanised by cervical dislocation.

### Whole Mount Alcian Blue/Alzarin Red Staining

PND5 skeletons were stained with Alcian Blue/Alzarin Red as previously described previously (43). Briefly, the right hind limb was separated before staining and fixed in 70% ethanol for μCT analysis. After removing other soft tissue, the skeleton was fixed and dehydrated in 90% ethanol for 7 days, with ethanol being changed on the 3rd and 5th day. Following this the skeleton was stained in freshly prepared Alcian blue solution (20mg Alcian blue 8GX in 22ml distilled water, 160 ml 100% ethanol and 40ml glacial acetic acid) for 3 days at room temperature. This was followed by sequential rehydration that with 70% ethanol treatment for 4-6 hours (ethanol change at 2-3 hours), followed by 40% ethanol for 4-6 hours (ethanol change at 2-3 hours), followed by 15% ethanol for 4-6 hours (ethanol change at 2-3 hours) and finally in water until the skeleton sank to the bottom of the staining container. After rehydration, skeletons were treated with freshly prepared 1% KOH for 1-2 days at room temperature until the remaining soft tissue became transparent. Skeletons were then stained with freshly prepared Alzarin Red S solution (1mg/100ml 1% KOH) for 3 days (staining solution changed every day). Finally, the skeletons were again treated with freshly prepared 1% KOH solution for 9hr hours at room temperature (KOH solution changed at every third hour). Skeletons were photographed and stored in 100% glycerol.

### Micro Computed Tomography (μCT) Analysis

μCT analysis was performed in a SkyScan 1172 Desktop X-ray μCT system. Neonatal femurs and tibiae were scanned at a spatial resolution of 10μm, whereas adult whole bones were scanned at a resolution of 17μm. Adult cortical and trabecular bone regions in the proximal tibiae were scanned at a resolution of 4.5μm. A 0.5mm aluminium filter and medium-pixel camera was used only for adult bones. Scanned image datasets were then reconstructed in NRecon^®^ (Version 1.4.1.0). The grey scale image datasets generated for individual bone was then analysed by interpolating regions of interest in CTAnalyser (Version 1.7.0.5) software. 3D skeletal models were rendered using Voxler 1.1, Golden software™. μCT-derived bone morphometric measurement symbols and units are presented in accordance with agreed guidelines (28). For μCT analysis of the response of bones to mechanical loading, we chose the same region of interest in the proximal tibial cortex as has been described previously by ourselves and others (29–31). Briefly the ROI was 1mm thick and extended from 1mm below the proximal tibial growth plate distally. We assessed the volume of new bone formed on the periosteal cortex, expressed as a proportion of the total original cortical volume.

### Dynamic Histomorphometry

Eight-week-old WT and *Ramp3^-/-^* mice were injected with calcein (100mg/kg) twice, one week apart before euthanasia. Animals were killed the day after the second label had been administered. Left tibiae were excised from mice and fixed in 70% ethanol prior to resin embedding and sectioning. Six longitudinal midshaft sections, 3μm apart, were analysed per specimen for each experiment. Quantification of the periosteal bone apposition rate was performed in 12, 300μm x 300μm regions (6 on the medial and 6 on the lateral side) per sample, using the OsteoMeasure^™^ system (OsteoMetrics^®^) using a Leica DMRB microscope. The detailed measurement protocol is described in the **Supplementary Methods** document.

### Haematoxylin and Eosin (H&E) and Tartrate-Resistant Acid Phosphatase (TRAP) Sample Processing

Right tibiae of eight-week-old WT and *Ramp3^-/-^* mice were fixed in ice cold 4% paraformaldehyde immediately after killing the animals. Bones were then decalcified and embedded in paraffin wax blocks before sectioning and staining. Briefly bones were decalcified in EDTA at room temperature for 4 weeks using 10-20 times volume of EDTA to volume of bone. The EDTA solution was changed each week. On completion of decalcification process, bones were embedded in paraffin wax and sectioned. Out of the six longitudinal mid sections 3µm apart, three alternate sections were used for H&E staining and the other three sections were used for TRAP staining. Haematoxylin and Eosin (H&E) (44) stained sections were used to study the bone versus cartilage differences. These sections were also used to determine the differences between the ratio of proliferative to hypertrophic zones in the growth plates. Tartrate-resistant acid phosphatase (TRAP) (45) stained sections were used to determine the number of osteoblast and osteoclasts and the osteoblast-osteoclast coverage. These measurements were performed on both the endocortical and the trabecular surface of tibia. The stained bone sections were scanned in the ScanScope^®^ (Aperio^®^) scanner. All histological analyses were then performed manually on scanned electronic sections in ImageScope^™^ (Aperio^®^), the e-slide ‘viewing’ software.

### Osteoblast and Osteoclast Numbers

We measured the number of osteoblasts, osteoblast covered bone surface, number of osteoclasts, osteoclast covered bone surface and trabecular and endocortical bone perimeters on both the endocortical and the trabecular bone surfaces in sections stained with H&E as described previously (**Supplementary Methods**). Cells and bone perimeters were manually marked on the ImageScope^™^ viewing software. The endocortical measurements were performed in 12 regions measuring 300μm x 300μm (6 on the medial and 6 on the lateral side), whereas trabecular measurements were performed in a 750μm x 750μm area. An offset of 300μm from the growth-plate was maintained in both endocortical and trabecular measurements. The detailed measurement protocol is described in the **Supplementary Methods** document.

### Growth Plate Analysis

Scanned H&E stained tibia sections were used for growth plate analysis. Number of proliferative cells, number of hypertrophic cells and the ratio of proliferative zone to hypertrophic zone were determined by manually marking the cells in an area of 1mm x 0.6mm mid growth plate region of each section. First, individual chondrocyte columns were manually marked as each column represents a clonal expansion of stem cells. Finally, the extent of proliferative zone and hypertrophic zone within each column was marked. The software then calculated the number of cells and proliferative cells to hypertrophic cells (PC/HC) ratio. The detailed measurement protocol is described in the **Supplementary Methods** document.

### Mechanical Loading

Groups of 6 male WT and 6 *Ramp3^-/-^* mice (17 weeks old at the start of the experiment) were subjected to mechanical loading of the left tibiae on 6 occasions over two weeks as previously described (46). Briefly, the flexed stifle (knee) and hock (ankle) joints were placed between domed cups of a mechanical loading device (Model 8511, Instron High Wycombe, UK) and held in place by a compressive force of 0.5N. The machine then applied axial compressive force of 13N, sufficient to induce 2,000 microstrain at over 100,000 microstrain sec^-1^ (the equivalent of human tibial bone strain on landing from a jump) (47). The load was then reduced at the same rate to 0.5N after 0.2 seconds, where it remained for 10 seconds before repeating. 40 of these ramped square wave loading cycles were applied on each loading day, occupying just over 6 minutes on each occasion. Bones were loaded on successive Mondays, Wednesdays and Fridays of 2 weeks and the mice injected with the fluorochrome calcein green (Sigma^®^) 10mgkg^-1^ on the first and last day of loading as previously described (46, 47) and the animals were killed on 3 days later and the left and right tibiae collected for μCT analysis and stored as before.

To analyse the amount of new bone formed in response to the mechanical loading, we defined the boundaries of the original periosteal cortex, which was composed of dense lamellar bone and measured the amount of woven bone formed outside of that margin. The position of the line determining the original periosteal cortex was checked on every third slice through the region of interest, and the volume of bone expressed in mm^3^.

### Primary Osteoblast Cultures

Calvariae were excised immediately after culling the animals and cells were collected from spun down fractions of sequential digestions. Each of the primary osteoblast cultures was established from three postnatal day 3 male mice. Excised calvariae were washed in Hank’s balanced salt solution containing calcium and magnesium without phenol red (Lonza^®^) and digested 5ml Collagenase 1A (Sigma^®^) (1mg/ml prepared in Hank’s solution) for 15 min digestion. Each cell fraction was pelleted and re-suspended in fresh alpha Minimum Essential Media (MEM) (Gibco^®^) and stored in ice. First fraction of cells was collected from the supernatant of a 30 min Collagenase 1A digestion at 37°C on a shaker at 200rpm. The calvariae were washed in PBS before the digestion for second cell fraction. The second cell fraction was obtained from the supernatant of 4mM tetrasodium EDTA pH7.0 digestion at 37°C on a shaker at 200rpm for 15 min. The calvariae were washed in Hank’s solution before the digestion for third cell fraction. The second cell fraction was obtained from the supernatant of a 30 min Collagenase 1A digestion at 37°C on a shaker at 200rpm. Finally, all the three cell fractions were pooled together and seeded in T75 culture flasks in alpha-MEM containing 10% heat inactivated fetal calf serum (FCS) (Gibco^®^) and 0.5% penicillin-streptomycin solution. On the third day when the flasks reached 80% confluency, cells were trypsinized (Trypsin-EDTA solution Gibco^®^) and re-seeded in either in 6 well culture plates (Nunc™) or T25 culture flasks and allowed to grow for three days prior to switching them to differentiation medium containing 5mM/l β-Glycerophosphate, 10nM dexamethasone and 100μg/ml Ascorbic acid. Cells were treated with DKK1 or Wnt3A or both on 20th day of differentiation prior to being harvested.

### Quantitative PCR (Q-PCR)

Double dye probe Q-PCR was performed with custom made FAM labelled primers designed by Primerdesign^®^ for each of the mouse RAMP (1, 2 and 3) genes. Primer sequences used RAMP1f 5’ACCTGGGATTTATAAGCCTGTTTA3’, RAMP1r 3’CATTTTTCCTCTGTCTCTTCTTCAT5’, RAMP2f 5’CCAACTGCTCCCTGGTGC3’, RAMP2r 3’GGAAGGGGATGAGGCAGATG5’, RAMP3f 5’CCAACTGCACCGAGATGGAG3’, and RAMP3r 3 ‘GGAGAAGAACTGCCTGTGGAT5’. Primers were validated, a copy number positive control was provided for each assay by Primerdesign^®^. Optimal number of reference genes and the most suitable reference genes were identified using the mouse geNorm^™^. The expression of each gene of interest was normalised to both 18s and β-actin (ACTB) in our assays. Each sample was run in triplicates per gene, per time point, per assay, per reference gene. Assays were run and analysed in Aplied Biosystems 7900 Real Time PCR machine using the absorbance maximum of 492nm and emission maximum of 517nm (for FAM detection). Thermo-cycler conditions used: initial 95°C for 10 minutes followed by 40 cycles of: ‘95°C for 15 seconds followed by 60°C for 60 seconds. Q-PCR reaction mix (final volume of 20μL):10μL Precision qPCR Mastermix (1X), 1μL primer/probe mix (primer concentration: 6pmols and probe concentration: 3pmols), 25ng cDNA template and RNAse/DNAse free water. The delta-delta CT value was calculated and normalised.

### Western Blotting

All protein samples were denatured in 4X Laemmli sample buffer, containing β-mercaptoethanol (Sigma^®^,Poole, UK), by boiling at 100°C for 5 minutes prior to electrophoresis. 100μg of protein per sample was electrophoresed on a 7% SDS polyacrylamide gel, and electro-blotted over to a PVDF membrane Hybond™. The membrane was incubated in blocking buffer- tris buffered saline tween (TBST) containing 5% BSA, for 1hr at room temperature. After blocking, the membranes were washed in TBST twice and incubated in primary antibody against β-catenin (Abcam^®^, ab27798), at a 1:200 dilution and β-actin (Abcam^®^, ab16039-500) at a 1:5000 dilution made up in blocking solution at 40C overnight, on rollers. The primary antibody was washed three times TBST and the membrane was incubated with Anti-rabbit-HRP (DAKO^®^ # P04498) secondary antibody, at a 1:5000 dilution made-up in blocking solution for 1 hour at room temperature. The biotinylated markers were incubated in TBST containing HRP-anti biotin for 1 hour at room temperature. The membranes were washed again as above, and incubated with chemiluminescent substrate, ECL Plus (Pierce^®^, Rockford Illinois, USA) for 5 min before exposing to photographic film. Films were exposed for 1min before developing.

### Statistical Analysis

Skeletal analysis: for comparisons between animals, μCT data was analysed using an unpaired two tailed Student’s T- test with 95% confidence interval and P value < 0.05 was considered significant. For comparisons between the left (loaded) and the right (non-loaded) tibia within each individual animal, a paired t-test was used. Protein expression and qPCR: All the datasets were analysed using two way-ANOVA test followed by Bonferroni’s post multiple test correction in GraphPad Prism^®^ 6. P value ≤ 0.05 was considered significant. For graphical representation we used GraphPad Prism^®^ 6.

## Results

### Gross Enhanced Skeletal Phenotype in Young *Ramp3^-/-^* Mice

**Figure 1:**
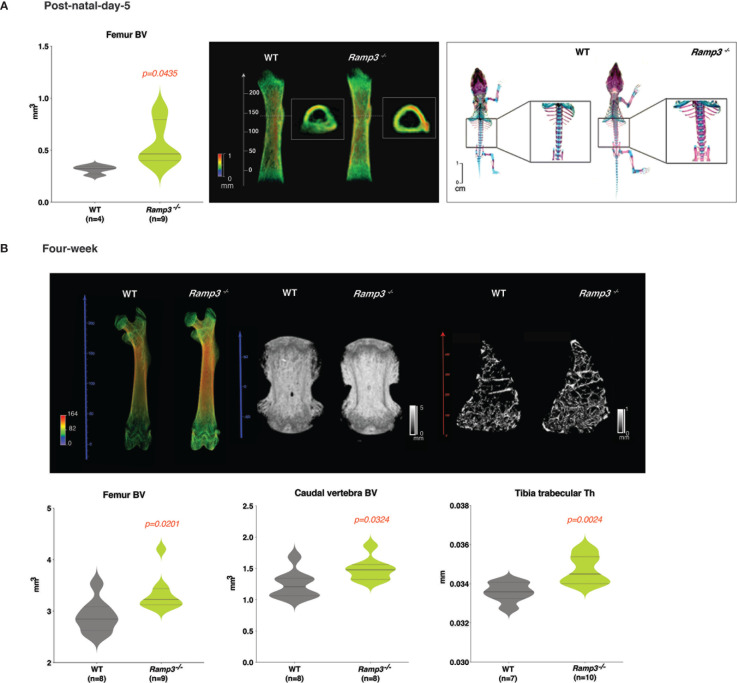
**(A)** MicroCT and skeletal staining reveals an advanced skeletal phenotype in the post-natal day 5 *Ramp3* ^-/-^ mice. Significant increase in the femur BV in *Ramp3* ^-/-^ mice (left) evidently observed in the 3D rendering of the femur bone (middle) (heat map scale: blue = low, red=high). Skeletal staining (right) reveals a remarkable increase in the bone formation in the vertebrae and long-bones in *Ramp3* ^-/-^ mice. **(B)** MicroCT reveals increase in whole femur and caudal vertebral bone volume in both the 3D bone renderings (top) and quantitative measurements (bottom) at 4 weeks of age. Trabecular analysis at a higher resolution identifies an increase in the trabecular thickness in the tibiae in both, the 3D models (top) and quantifications (bottom) in the *Ramp3* ^-/-^ mice. (Heat map scales in 3D models: Coloured scale- blue=low and red=high; Black and White scale – black = low and white= high).

Both µCT and skeletal staining suggested a consistent increase in bone volume (p=0.044) *in Ramp3^-/-^* post-natal day-5 (**Figure 1A** and **Supplementary Table 1**). The increase in the bone volume was indeed due to the advanced bone formation in the *Ramp3^-/-^* mice as revealed by densitometric three-dimensional models of single bones rendered from µCT scans and skeletal staining the extent of ossification in long bones and the vertebrae the post-natal day-5 *Ramp3^-/-^* mice (**Figure 1B**). The enhanced femur bone volume (BV) (p=0.02) and advanced ossification and increased bone volume in the vertebrae (p=0.032) were consistent in the *Ramp3^-/-^* mice even at 4 weeks of age (**Figure 1B**, and **Supplementary Table 1**). Furthermore, *Ramp3^-/-^* mice at 4 weeks of age also exhibited increased trabecular thickness (Th) in both femurs and the tibiae (p=0.02) which were remarkably evident in the three-dimensional renderings generated from the scans (**Figure 1B** and **Supplementary Tables 1**, **2**). These profound differences in the skeletal phenotype in the *Ramp3^-/-^* mice corroborated with our observations *in vitro*, wherein, the reduced *Ramp3* expression associated with progression of osteoblast differentiation, further providing evidence to the role of RAMP3 as a negative regulator of skeletal bone mass.

### Characteristic Age Dependent Skeletal Phenotype in *Ramp3^-/-^* Mice

**Figure 2:**
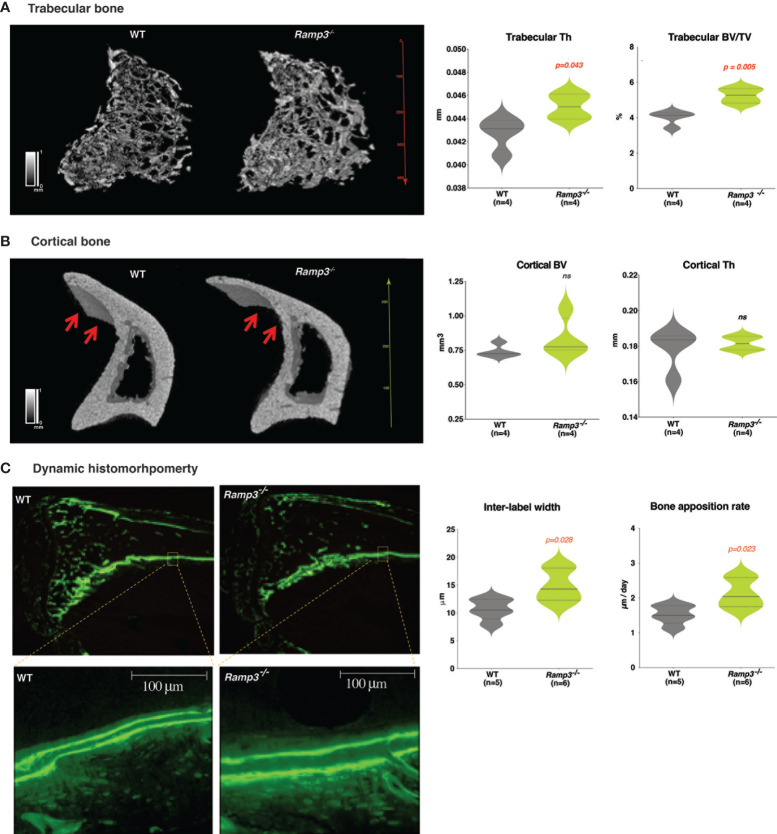
**(A)** Increase in the trabecular bone thickness and the bone volume/tissue volume ratio revealed through the 3D rendering of the microCT scan (left) and quantitative analysis (right) in *Ramp3* ^-/-^ mice at 8 weeks of age. **(B)** Structural differences such as the thinner and more elongated tibial crest (red arrows) observed in the tibia cortical bone in the *Ramp3* ^-/-^ mice at 8 weeks. Despite the thinner appearance of the cortical bone in the 3D model, the quantitative analysis does not suggest a decrease in the overall cortical bone volume in the *Ramp3* ^-/-^ mice. (Black and white scale in the models: black =low, white=high). **(C)** Calcein-labelled tibia sections demonstrating dynamic histomorphometry. *Ramp3* ^-/-^ bones have increased distance between the two successive calcein labels in the cortical bone (left). Significant increase in the inter-label width and bone apposition rate in the *Ramp3* ^-/-^ mice detected from the dynamic histomorphometry quantifications (right). ns, Not significant.

At eight weeks of age, we observed distinct structural changes in the long bones of the *Ramp3^-/-^* mice. As expected, *Ramp3^-/-^* mice maintained the increase in the trabecular thickness (Th) (p=0.04) and a complementary bone volume to tissue volume ratio (BV/TV) (p=0.005) (**Figure 2A** and **Supplementary Tables 1**, **2**). However, three-dimensional renderings of the µCT scans of proximal tibiae revealed longer and thinner tibial crest in the *Ramp3^-/-^* mice and there were no significantly quantifiable differences in the bone volume (BV) and thickness (Th) of the cortical bone by µCT (**Figure 2B** and **Supplementary Tables 1**, **2**). To verify if there was any difference in bone formation, we performed dynamic histomorphometry. Indeed, the *Ramp3^-/-^* mice had significant increases in bone apposition rate (p=0.023) revealed by the increased inter-label distance (p=0.028) suggestive of a higher rate of bone formation in periosteal tibia cortical bone in these mice (**Figure 2C**).

### Increase in the Bone Formation in Response to Mechanical Loading in *Ramp3^-/-^* Mice

**Figure 3:**
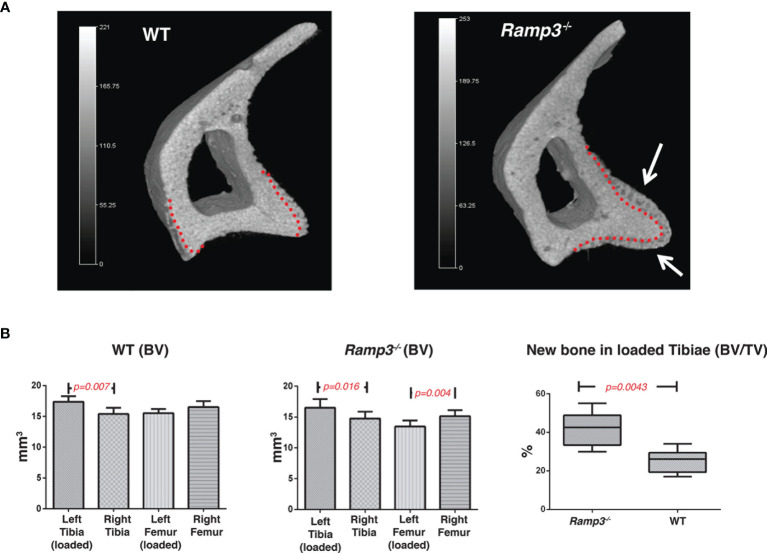
**(A)** 3D renderings of a representative tibia cortical bone of a loaded *Ramp3* ^-/-^ and WT mice. The red dotted lines represent the original cortical boundary between the dense lamellar cortical bone and the newly formed woven bone. The white arrows indicate the significantly increased adaptive bone formation in the *Ramp3* ^-/-^ compared with the WT mice. Grey scale represents the size and bone density gradient. n=6 each group. **(B)** Micro-CT volumetric measurements identify differences loaded and unloaded bones in the WT (left) and *Ramp3* ^-/-^ (middle) mice. Significant increase in the bone volume/tissue volume ratio of the loaded *Ramp3* ^-/-^ bone suggest a significant increase in the new bone formation in the *Ramp3* ^-/-^ compared to the WT (right) in response to loading.

The differences in architecture of the tibial crest at 8 weeks of age were suggestive of altered mechanical load experienced by the *Ramp3^-/-^* mice as the region is where the patellar ligament inserts, providing for weight-bearing function by extension of the stifle (knee) joint. Using µCT to measure the volume of new form bone in response to loading regimen at 17 weeks of age, we determined the differences within the WT and *Ramp3^-/-^* cohorts. Both the WT and *Ramp3^-/-^* mice exhibited an adaptive response to the loading in the loaded tibia. In WTs, the bone formed in response to loading in the region of interest in the proximal tibia significantly increased by 0.12mm^3^ ± SEM 0.015 (**Figures 3A, B**). However, there was a greater bone formation in response to loading the *Ramp3^-/-^* mice 0.16 mm^3^ ± SEM 0.01 which represents an increase in the volume of bone formed as a result of the loading of nearly 30% providing a strong functional evidence of an advanced skeletal phenotype in the *Ramp3^-/-^* mice (**Figures 3A, B**).

### Increase in the Osteoblast Numbers and Growth Plate Thickness in *Ramp3^-/-^* Mice

**Figure 4:**
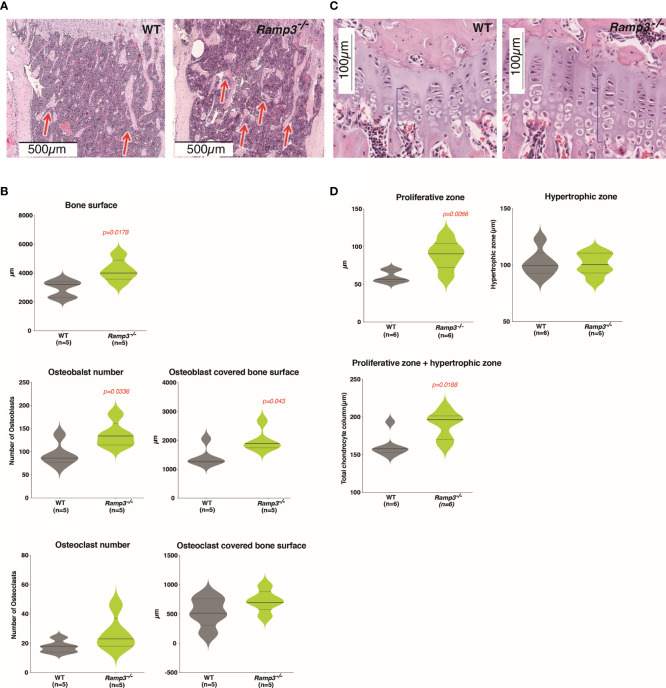
**(A)** Haematoxylin and eosin-stained tibia sections reveal increased trabecular bone (red arrows) in the *Ramp3* ^-/-^ mice at 8 weeks of age. **(B)** Histological measurements identify increase in the bone surface, osteoblast number and osteoblast covered bone surface (top) in the *Ramp3* ^-/-^ mice. No differences in the osteoclast numbers and osteoclast covered bone surface (bottom) identified. **(C)** Haematoxylin and eosin-stained tibia sections of the growth plate show increase in the growth plate thickness (blue brace brackets). **(D)** Quantitative analysis of the growth plate reveals that the proliferative zone, but no the hypertrophic zone contributes to the gross increase in the growth plate thickness.

The subtle difference in the microarchitecture of the *Ramp3^-/-^* skeleton, associated with more bone formation, prompted us to perform histology to further examine the cell distribution in the skeletal niche of these regions of interest. Histology on H&E-stained longitudinal sections of the tibia revealed significant increases in the trabecular bone surface concurrent with our µCT data (**Figures 4A, B**). *Ramp3^-/-^* TRAP stained bone sections revealed significantly higher osteoblast number and a higher osteoblast covered bone surface in the trabecular bone compared to WTs (**Figure 4B**). Notably, there were no difference in osteoclast numbers or osteoclast covered bone surface (**Figure 4B**). Furthermore, the growth plates in the sections of *Ramp3^-/-^* tibiae presented larger chondrocyte columns with larger proliferative chondrocyte zone (**Figures 4C, D**). On the other hand, the hypertrophic zone was variable in both WT and *Ramp3^-/-^* mice (**Figure 4D**). The larger proliferative chondrocyte zone and inter-label distances (**Figure 2C**), not only reflect the advanced ossification in these mice at younger age, but also give insight into the skeletal niche accountable for an improved microarchitecture in the *Ramp3^-/-^* mice.

### Inverse Association Between the Ramp3 Gene Expression and Osteoblast Differentiation *In Vitro*

**Figure 5:**
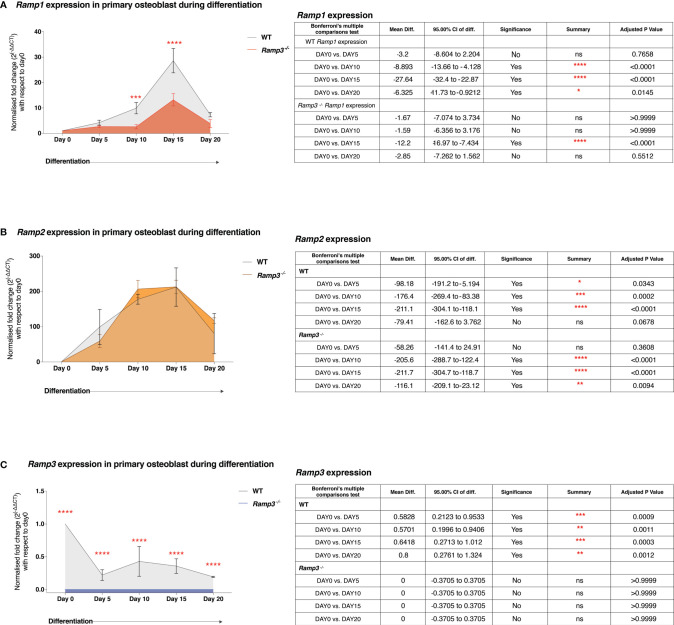
Quantitative PCR expression of *Ramp1* **(A)**, *Ramp2* **(B)** and *Ramp3* **(C)** in *Ramp3* ^-/-^ and WT differentiating osteoblast cultures. Level of significance for the difference in gene expression between the genotypes is indicated with the number of asterisks (adjusted p value 0.05=*, p value 0.001 = **, p value 0.0001 = ***, p value 0.00001 = ****). The tables adjacent to the plots summarise the mean differences, 95% confidence intervals and adjusted p value post ANOVA test and the *post-hoc* Bonferroni’s correction. ns, Not significant.

Several systemic hormones and local factors within the skeletal niche, induce/influence different signalling pathways within the cells of osteoprogenitor lineage during osteoblast differentiation. Since there was a specific influence on the osteoblasts and not osteoclasts number and coverage in bone (**Figures 4A, B**), we hypothesised that the expression of *Ramp* genes plays a crucial role in osteoblast differentiation. We determined the expression patterns of individual *Ramp* genes (Q-PCR), first and foremost in calvarial osteoblast cultures established from post-natal day-3 WT (129/SvEv) mice. We then looked at the difference in the expression patterns between the WT and the *Ramp3^-/-^* osteoblasts to determine any linked expression profile. We observed striking differences between the patterns of individual *Ramp* gene expression, in that *Ramp1/2* (**Figures 5A, B**) expression steady increased from day-0 to day-15 of the differentiation whist *Ramp3* expression declined with the progression of osteoblast differentiation (**Figure 5C**) in WT osteoblast cultures. However, despite the same pattern of expression, osteoblasts derived from *Ramp3^-/-^* mice had significantly lower *Ramp1* expression levels compared to the WT osteoblasts throughout the differentiation (**Figure 5A**) suggesting an association between *Ramp1 and 3* in the osteoblasts. Moreover, there were no differences in *Ramp2* expression levels between WT and *Ramp3^-/-^* mice (**Figure 1B**), which corroborates with reports by Dackor et al, that loss of *Ramp3* does not have a reciprocal effect on *Ramp2* expression (37). Indeed, *Ramp3* expression in *Ramp3^-/-^* osteoblasts was not quantifiable, hence verifying our model (**Figure 5C**). Given the complex pharmacology of the RAMPs, the selective modulated expression of *Ramp3* in osteoblasts could influence other signalling pathways fundamental to osteoblast differentiation such as the *Wnt* pathway, which is yet to be studied comprehensively. Our preliminary data on 3 independent osteoblast cultures, show increased total β-catenin expression in the *Ramp3* ^-/-^s (**Supplementary Figure 1**). These findings collectively suggest that reduced *Ramp3* expression associates with progression of osteoblast differentiation.

## Discussion

In this study we have shown that *Ramp3^-/-^* mice have a distinct bone phenotype characterised by accelerated bone development and growth and an increased response to mechanical loading when compared with WT animals of the same background strain. Specifically, we have established that in the WT osteoblasts, *Ramp3* expression is negatively associated with advancement in differentiation *in vitro* so that absence of RAMP3 accelerates osteoblast differentiation and consequently bone apposition. With increasing age, the gross high bone volume phenotype in the *Ramp3* ^-/-^ mice becomes less obvious but more complex with structural changes in the microarchitecture particularly in areas of the bone that could be expected to experience high levels of mechanical loading in life. This is exemplified in the tibial crest, whose proximal part is the point of attachment of the patellar ligament and so responsible for extension of the stifle (knee) joint, and therefore weight-bearing and propulsion in the hind limb. The increase in bone apposition rate and the growth plate thickness demonstrates the range of altered dynamic skeletal regulation in these mice but appears to be involved in the rate of bone lengthening rather than the amount, as *Ramp3^-/-^* mice did not have longer bones than WTs as adults. This complexity in the phenotype is consistent with complex RAMP pharmacology and the overall explanation of the phenotype of these mice is not simple. This is because RAMP3 governs ligand selectivity for both the calcitonin-like receptor (CLR) and the calcitonin receptor (CTR) (30, 32, 33, 48). For other receptors, RAMPs alter the consequence of ligand binding without altering receptor selectivity e.g. the glucagon receptor (RAMP3), vasoactive-intestinal peptide/pituitary adenylate cyclase-activating peptide 1 (VIP/VPAC1) receptor (RAMP1 and 3) (32, 49), GPR30 (RAMP3) (50) and a GPCR class III receptor – the calcium sensing receptor (CaSr) (RAMP1 and 3) (33, 51–53). Furthermore, RAMP3 interacts with many members of the chemokine/cytokine family of receptors, and in the context of atypical chemokine receptor 3 and the CLR, RAMP3 regulates the endosomal sorting and recycling of atypical chemokine receptors (54). As well as roles in forming the functional high affinity receptor for AM and AMY, RAMP3/CLR (AM2R) receptors also have appreciable affinity for CGRP which could contribute to functional roles in exciting emerging research on CGRP sensory nerves and the hematopoietic stem cell niche (55–57).

As the number of GPCRs known to interact with RAMPs is increasing, it becomes clear that the phenotype of these animals may be due to alterations in more than one receptor’s signalling pathways. It is certainly possible that *Ramp3* ^-/-^ mice have alterations in signalling *via* ADM-2 and AMY-3 receptors, but possibly other receptors, some which may remain to be discovered as RAMP partners. The role of RAMPs in regulating biasing of other GPCRs is poorly understood (32, 33, 58, 59). For example, the specific roles of the two ADM receptor variants are only becoming clear as a result of recent developments of useful tool compounds for the CLR/RAMP heteromers (60–62) and there is little information on the roles of the 3 separate AMY receptor types in physiology (30, 32, 33). Paradoxically though, both amylin and adrenomedullin are known to have positive effects on osteoblasts in culture and to induce bone formation *in vivo* so the effect of *Ramp3* deletion in increasing bone mass and development is not easily explained (25, 63–67) unless removal of RAMP3-mediated ADM and AMY effects allow anabolic effects of ADM1 and AMY1&2 receptors to dominate.

From the alterations in the cell populations in the proliferative zone in the growth plate, it is reasonable to assume that primitive stem cell populations such as the osteochondroprogenitor cells and not just osteoblast lineage committed stem cells could be influenced by *Ramp3* expression, and that there is a plausible association between *Ramp3* and early osteoblast differentiation players such as *Wnt*. In a study of chronological target gene expression, *Ramp3* is downregulated in the first 24 hours after *Wnt3A* stimulation, indicating that it is one of the early response genes targeted by *Wnt* (68). There is also evidence of phosphorylated β-catenin induction selectively down-regulating *Ramp3* but not *Ramp1/2* expression (69, 70). If *Ramp3* deficiency cooperates with the Wnt pathway, it could provide context to the cellular mechanisms that dictate the complex skeletal phenotype in the *Ramp3^-/-^* mice. Our preliminary data, where we observe increase in the total β-catenin expression in differentiating primary osteoblasts from *Ramp3^-/-^* fetal calvaria (**Supplementary Figure 1**), supports the association between *Wnt* and *Ramp3*. Certainly, recent report on enhanced osteogenic differentiation and pro-angiogenic potential of the bone marrow mesenchymal stem cells potential upon ADM2 treatment, *via* accumulation and activation of β-catenin, further supports the association of the *Wnt* signalling and the CT peptides (71). However, the specific mechanisms through which the *Ramp3-Wnt* interaction influence skeletal regulation require further research.

Taken together, there are clear implications of our findings. The evidence for increased bone mass and its various markers and the rate of bone development are consistent with increased osteoblast numbers and activity. Interestingly the lack of a parallel increase in osteoclasts and resorption indicates that the effect of RAMP3 is at a controlling level of skeletal physiology. Had the effects been due to focused stimulation of bone formation through direct actions on osteoblasts, then it would be expected that osteoclast numbers and activity would also increase to maintain normal skeletal mass and architecture. RAMP3 appears to affect some fundamental set point for skeletal properties in an anabolic way. One limitation of this study is that we did not have access to unlimited numbers of mice and in a few cases group sizes are small. That has effects on the strength that might be ascribed to the statistical analysis of the data we present, but we believe that further studies by others will increase our knowledge of the role of RAMP3 in the future.

The finding of this study raises other more general questions such as what is the purpose of a gene whose expression slows bone development and reduces bone mass? The answer is not certain, but it is likely that it links back to the way the skeleton evolved a homeostatic adaptive mechanism in the distant past. During vertebrate evolution, when some animals existed with very over-engineered skeletons, their individual resistance to trauma would be great, but their ability to catch prey or evade predators would be reduced, so that they would be outcompeted by animals with more tuned lower skeletal mass. Under those circumstances, management of optimum bone mass was weighted in favour of efficient and speedy locomotion (something that is almost entirely functionally irrelevant to humans today). Whether this means that therapeutic blockade of RAMP3 would have any consequence on bone mass is currently unclear, but it is a possible inference from our data.

## Data Availability Statement

The original contributions presented in the study are included in the article/**Supplementary Material**, further inquiries can be directed to the corresponding author.

## Ethics Statement

The animal study was reviewed and approved by Local Ethics Committee, University of Sheffield and UK Home Office.

## Author Contributions

TS, GR, and SP designed research. SP performed research and collected and analysed data. ML and NW performed the mechanical loading studies and collected and analysed those data. SP, GR, and TS wrote the manuscript. KC and MK generated the RAMP3^-/-^ mice. All authors critically reviewed the paper. All authors contributed to the article and approved the submitted version.

## Funding

This work was supported in part by grants from the Biotechnology and Biological Sciences Research Council (BB/ F008392/1, S18293/2, S17443/2) to fund colonies of WT and RAMP3-/- mice in Sheffield and by the US National Institutes of Health (NIDDK R01 DK119145) for the work described in this manuscript. Open Access funding was provided by the University of Sheffield.

## Conflict of Interest

TS and GR hold stock and positions in Modulus Oncology, a company developing ADM2 (CLR/RAMP3) receptor antagonists.

The remaining authors declare that the research was conducted in the absence of any commercial or financial relationships that could be construed as a potential conflict of interest.

## Publisher’s Note

All claims expressed in this article are solely those of the authors and do not necessarily represent those of their affiliated organizations, or those of the publisher, the editors and the reviewers. Any product that may be evaluated in this article, or claim that may be made by its manufacturer, is not guaranteed or endorsed by the publisher.

## Supplementary Material

Supplementary Figure 1 Protein expression of total β-catenin in differentiating primary osteoblasts. Representative western blot (top) showing increased beta-catenin expression (92kD) in *Ramp3* ^-/-^ primary osteoblast lysates compared to WTs at day 10,15 and 20 of differentiation. Western blotting was performed for each of the 3 independent osteoblast differentiation experiments. Densitometric analysis (bottom) of the western blots (n=3) confirmed the significance in differential expression of beta-catenin. Level of significance for the difference in gene expression between the genotypes was calculated using the ANOVA test and, is indicated with the number of asterisks (adjusted p value 0.05=*, p value 0.001 = ** so on and so forth).

Supplementary Table 1 Data from microCT analysis of Bones from WT and RAMP3 null mice - post natal day 5 and 4 weeks of age.

Supplementary Table 2 Data from microCT and histomorphometric analysis of Bones from WT and RAMP3 null mice – 8 weeks of age.

## References

[1] Camacho PM Petak SM Binkley N Clarke BL Harris ST Hurley DL. American Association of Clinical Endocrinologists and American College of Endocrinology Clinical Practice Guidelines for the Diagnosis and Treatment of Postmenopausal Osteoporosis - 2016. Endocr Pract (2016) 22(Suppl 4):1–42. doi: 10.4158/EP161435.GL 27662240

[2] Watts NB Adler RA Bilezikian JP Drake MT Eastell R Orwoll ES. Osteoporosis in Men: An Endocrine Society Clinical Practice Guideline. J Clin Endocrinol Metab (2012) 97(6):1802–22. doi: 10.1210/jc.2011-3045 22675062

[3] Buckley L Guyatt G Fink HA Cannon M Grossman J Hansen KE. 2017 American College of Rheumatology Guideline for the Prevention and Treatment of Glucocorticoid-Induced Osteoporosis. Arthritis Rheumatol (2017) 69(8):1521–37. doi: 10.1002/art.40137 28585373

[4] Ettinger B Black DM Mitlak BH Knickerbocker RK Nickelsen T Genant HK. Reduction of Vertebral Fracture Risk in Postmenopausal Women With Osteoporosis Treated With Raloxifene: Results From a 3-Year Randomized Clinical Trial. Multiple Outcomes of Raloxifene Evaluation (MORE) Investigators. JAMA (1999) 282(7):637–45. doi: 10.1001/jama.282.7.637 10517716

[5] Silverman SL Christiansen C Genant HK Vukicevic S Zanchetta JR de Villiers TJ. Efficacy of Bazedoxifene in Reducing New Vertebral Fracture Risk in Postmenopausal Women With Osteoporosis: Results From a 3-Year, Randomized, Placebo-, and Active-Controlled Clinical Trial. J Bone Miner Res (2008) 23(12):1923–34. doi: 10.1359/jbmr.080710 18665787

[6] Smith MR Saad F Coleman R Shore N Fizazi K Tombal B. Denosumab and Bone-Metastasis-Free Survival in Men With Castration-Resistant Prostate Cancer: Results of a Phase 3, Randomised, Placebo-Controlled Trial. Lancet (2012) 379(9810):39–46. doi: 10.1016/S0140-6736(11)61226-9 22093187 PMC3671878

[7] Martin TJ. Parathyroid Hormone-Related Protein, Its Regulation of Cartilage and Bone Development, and Role in Treating Bone Diseases. Physiol Rev (2016) 96(3):831–71. doi: 10.1152/physrev.00031.2015 27142453

[8] Appelman-Dijkstra NM Papapoulos SE. Clinical Advantages and Disadvantages of Anabolic Bone Therapies Targeting the WNT Pathway. Nat Rev Endocrinol (2018) 14(10):605–23. doi: 10.1038/s41574-018-0087-0 30181608

[9] Neer RM Arnaud CD Zanchetta JR Prince R Gaich GA Reginster JY. Effect of Parathyroid Hormone (1-34) on Fractures and Bone Mineral Density in Postmenopausal Women With Osteoporosis. N Engl J Med (2001) 344(19):1434–41. doi: 10.1056/NEJM200105103441904 11346808

[10] Khosla S Hofbauer LC. Osteoporosis Treatment: Recent Developments and Ongoing Challenges. Lancet Diabetes Endocrinol (2017) 5(11):898–907. doi: 10.1016/S2213-8587(17)30188-2 28689769 PMC5798872

[11] Khosla S Shane E. A Crisis in the Treatment of Osteoporosis. J Bone Miner Res (2016) 31(8):1485–7. doi: 10.1002/jbmr.2888 27335158

[12] Pietrogrande L Raimondo E. Abaloparatide for the Treatment of Postmenopausal Osteoporosis. Drugs Today (Barc) (2018) 54(5):293–303. doi: 10.1358/dot.2018.54.5.2800621 29911694

[13] Jolette J Attalla B Varela A Long GG Mellal N Trimm S. Comparing the Incidence of Bone Tumors in Rats Chronically Exposed to the Selective PTH Type 1 Receptor Agonist Abaloparatide or PTH(1-34). Regul Toxicol Pharmacol (2017) 86:356–65. doi: 10.1016/j.yrtph.2017.04.001 28389324

[14] Vahle JL Long GG Sandusky G Westmore M Ma YL Sato M. Bone Neoplasms in F344 Rats Given Teriparatide [rhPTH(1-34)] Are Dependent on Duration of Treatment and Dose. Toxicol Pathol (2004) 32(4):426–38. doi: 10.1080/01926230490462138 15204966

[15] Jolette J Wilker CE Smith SY Doyle N Hardisty JF Metcalfe AJ. Defining a Noncarcinogenic Dose of Recombinant Human Parathyroid Hormone 1-84 in a 2-Year Study in Fischer 344 Rats. Toxicol Pathol (2006) 34(7):929–40. doi: 10.1080/01926230601072301 17178693

[16] Michelangeli VP Fletcher AE Allan EH Nicholson GC Martin TJ. Effects of Calcitonin Gene-Related Peptide on Cyclic AMP Formation in Chicken, Rat, and Mouse Bone Cells. J Bone Miner Res (1989) 4(2):269–72. doi: 10.1002/jbmr.5650040220 2543186

[17] Takahashi N Yamana H Yoshiki S Roodman GD Mundy GR Jones SJ. Osteoclast-Like Cell Formation and Its Regulation by Osteotropic Hormones in Mouse Bone Marrow Cultures. Endocrinology (1988) 122(4):1373–82. doi: 10.1210/endo-122-4-1373 3345718

[18] Zaidi M Moonga BS Abe E. Calcitonin and Bone Formation: A Knockout Full of Surprises. J Clin Invest (2002) 110(12):1769–71. doi: 10.1172/JCI200217425 PMC151662 12488426

[19] Millet I Vignery A. The Neuropeptide Calcitonin Gene-Related Peptide Inhibits TNF-Alpha But Poorly Induces IL-6 Production by Fetal Rat Osteoblasts. Cytokine (1997) 9(12):999–1007. doi: 10.1006/cyto.1997.0245 9417811

[20] Sakagami Y Girasole G Yu XP Boswell HS Manolagas SC. Stimulation of Interleukin-6 Production by Either Calcitonin Gene-Related Peptide or Parathyroid Hormone in Two Phenotypically Distinct Bone Marrow-Derived Murine Stromal Cell Lines. J Bone Miner Res (1993) 8(7):811–6. doi: 10.1002/jbmr.5650080706 8394639

[21] Shih C Bernard GW. Calcitonin Gene Related Peptide Enhances Bone Colony Development *In Vitro* . Clin Orthop Relat Res (1997) 334:335–44. doi: 10.1097/00003086-199701000-00043 9005931

[22] Cornish J Naot D Reid IR. Adrenomedullin–A Regulator of Bone Formation. Regul Pept (2003) 112(1-3):79–86. doi: 10.1016/S0167-0115(03)00025-9 12667628

[23] Cornish J Callon KE Coy DH Jiang NY Xiao L Cooper GJ. Adrenomedullin Is a Potent Stimulator of Osteoblastic Activity *In Vitro* and *In Vivo* . Am J Physiol (1997) 273(6):E1113–20. doi: 10.1152/ajpendo.1997.273.6.E1113 9435526

[24] Kano H Kohno M Yasunari K Yokokawa K Horio T Ikeda M. Adrenomedullin as a Novel Antiproliferative Factor of Vascular Smooth Muscle Cells. J Hypertens (1996) 14(2):209–13. doi: 10.1097/00004872-199602000-00009 8728298

[25] Cornish J Reid IR. Effects of Amylin and Adrenomedullin on the Skeleton. J Musculoskelet Neuronal Interact (2001) 2(1):15–24. 15758473

[26] Granholm S Lundberg P Lerner UH. Expression of the Calcitonin Receptor, Calcitonin Receptor-Like Receptor, and Receptor Activity Modifying Proteins During Osteoclast Differentiation. J Cell Biochem (2008) 104(3):920–33. doi: 10.1002/jcb.21674 18384073

[27] Wang F Kong L Wang W Shi L Wang M Chai Y. Adrenomedullin 2 Improves Bone Regeneration in Type 1 Diabetic Rats by Restoring Imbalanced Macrophage Polarization and Impaired Osteogenesis. Stem Cell Res Ther (2021) 12(1):288. doi: 10.1186/s13287-021-02368-9 33985585 PMC8117361

[28] Liang YL Khoshouei M Deganutti G Glukhova A Koole C Peat TS. Cryo-EM Structure of the Active, Gs-Protein Complexed, Human CGRP Receptor. Nature (2018) 561(7724):492–7. doi: 10.1038/s41586-018-0535-y PMC6166790 30209400

[29] Mackie DI Al Mutairi F Davis RB Kechele DO Nielsen NR Snyder JC. hCALCRL Mutation Causes Autosomal Recessive Nonimmune Hydrops Fetalis With Lymphatic Dysplasia. J Exp Med (2018) 215(9):2339–53. doi: 10.1084/jem.20180528 PMC6122977 30115739

[30] McLatchie LM Fraser NJ Main MJ Wise A Brown J Thompson N. RAMPs Regulate the Transport and Ligand Specificity of the Calcitonin-Receptor-Like Receptor. Nature (1998) 393(6683):333–9. doi: 10.1038/30666 9620797

[31] Hay DL Chen S Lutz TA Parkes DG Roth JD. Amylin: Pharmacology, Physiology, and Clinical Potential. Pharmacol Rev (2015) 67(3):564–600. doi: 10.1124/pr.115.010629 26071095

[32] Christopoulos G Perry KJ Morfis M Tilakaratne N Gao Y Fraser NJ. Multiple Amylin Receptors Arise From Receptor Activity-Modifying Protein Interaction With the Calcitonin Receptor Gene Product. Mol Pharmacol (1999) 56(1):235–42. doi: 10.1124/mol.56.1.235 10385705

[33] Christopoulos A Christopoulos G Morfis M Udawela M Laburthe M Couvineau A. Novel Receptor Partners and Function of Receptor Activity-Modifying Proteins. J Biol Chem (2003) 278(5):3293–7. doi: 10.1074/jbc.C200629200 12446722

[34] Booe JM Walker CS Barwell J Kuteyi G Simms J Jamaluddin MA. Structural Basis for Receptor Activity-Modifying Protein-Dependent Selective Peptide Recognition by a G Protein-Coupled Receptor. Mol Cell (2015) 58(6):1040–52. doi: 10.1016/j.molcel.2015.04.018 PMC4504005 25982113

[35] JG J Simms J Barwell J Poyner DR Watkins HA Pioszak AA. An Allosteric Role for Receptor Activity-Modifying Proteins in Defining GPCR Pharmacology. Cell Discov (2016) 2:16012. doi: 10.1038/celldisc.2016.12 27462459 PMC4869360

[36] Tsujikawa K Yayama K Hayashi T Matsushita H Yamaguchi T Shigeno T. Hypertension and Dysregulated Proinflammatory Cytokine Production in Receptor Activity-Modifying Protein 1-Deficient Mice. Proc Natl Acad Sci USA (2007) 104(42):16702–7. doi: 10.1073/pnas.0705974104 PMC2034234 17923674

[37] Dackor R Fritz-Six K Smithies O Caron K. Receptor Activity-Modifying Proteins 2 and 3 Have Distinct Physiological Functions From Embryogenesis to Old Age. J Biol Chem (2007) 282(25):18094–9. doi: 10.1074/jbc.M703544200 17470425

[38] Caron KM Smithies O. Extreme Hydrops Fetalis and Cardiovascular Abnormalities in Mice Lacking a Functional Adrenomedullin Gene. Proc Natl Acad Sci USA (2001) 98(2):615–9. doi: 10.1073/pnas.98.2.615 PMC14636 11149956

[39] Dackor RT Fritz-Six K Dunworth WP Gibbons CL Smithies O Caron KM. Hydrops Fetalis, Cardiovascular Defects, and Embryonic Lethality in Mice Lacking the Calcitonin Receptor-Like Receptor Gene. Mol Cell Biol (2006) 26(7):2511–8. doi: 10.1128/MCB.26.7.2511-2518.2006 PMC1430335 16537897

[40] Ichikawa-Shindo Y Sakurai T Kamiyoshi A Kawate H Iinuma N Yoshizawa T. The GPCR Modulator Protein RAMP2 Is Essential for Angiogenesis and Vascular Integrity. J Clin Invest (2008) 118(1):29–39. doi: 10.1172/JCI33022 18097473 PMC2147670

[41] Dackor R Caron K. Mice Heterozygous for Adrenomedullin Exhibit a More Extreme Inflammatory Response to Endotoxin-Induced Septic Shock. Peptides (2007) 28(11):2164–70. doi: 10.1016/j.peptides.2007.08.012 PMC2121581 17889965

[42] Kadmiel M Fritz-Six K Pacharne S Richards GO Li M Skerry TM. Research Resource: Haploinsufficiency of Receptor Activity-Modifying Protein-2 (RAMP2) Causes Reduced Fertility, Hyperprolactinemia, Skeletal Abnormalities, and Endocrine Dysfunction in Mice. Mol Endocrinol (2011) 25(7):1244–53. doi: 10.1210/me.2010-0400 PMC3125095 21566080

[43] Gomez C David V Peet NM Vico L Chenu C Malaval L. Absence of Mechanical Loading *In Utero* Influences Bone Mass and Architecture But Not Innervation in Myod-Myf5-Deficient Mice. J Anat (2007) 210(3):259–71. doi: 10.1111/j.1469-7580.2007.00698.x PMC2100282 17331176

[44] Cardiff RD Miller CH Munn RJ. Manual Hematoxylin and Eosin Staining of Mouse Tissue Sections. Cold Spring Harb Protoc (2014) 2014(6):655–8. doi: 10.1101/pdb.prot073411 24890205

[45] Yang J Bi X Li M. Osteoclast Differentiation Assay. Methods Mol Biol (2019) 1882:143–8. doi: 10.1007/978-1-4939-8879-2_12 30378050

[46] Wang N Rumney RM Yang L Robaye B Boeynaems JM Skerry TM. The P2Y13 Receptor Regulates Extracellular ATP Metabolism and the Osteogenic Response to Mechanical Loading. J Bone Miner Res (2013) 28(6):1446–56. doi: 10.1002/jbmr.1877 23362109

[47] Hillam RA Goodship AE Skerry TM. Peak Strain Magnitudes and Rates in the Tibia Exceed Greatly Those in the Skull: An *In Vivo* Study in a Human Subject. J Biomech (2015) 48(12):3292–8. doi: 10.1016/j.jbiomech.2015.06.021 PMC4601046 26232812

[48] Muff R Buhlmann N Fischer JA Born W. An Amylin Receptor Is Revealed Following Co-Transfection of a Calcitonin Receptor With Receptor Activity Modifying Proteins-1 or -3. Endocrinology (1999) 140(6):2924–7. doi: 10.1210/endo.140.6.6930 10342886

[49] Wootten D Lindmark H Kadmiel M Willcockson H Caron KM Barwell J. Receptor Activity Modifying Proteins (RAMPs) Interact With the VPAC2 Receptor and CRF1 Receptors and Modulate Their Function. Br J Pharmacol (2013) 168(4):822–34. doi: 10.1111/j.1476-5381.2012.02202.x PMC3631373 22946657

[50] Lenhart PM Broselid S Barrick CJ Leeb-Lundberg LM Caron KM. G-Protein-Coupled Receptor 30 Interacts With Receptor Activity-Modifying Protein 3 and Confers Sex-Dependent Cardioprotection. J Mol Endocrinol (2013) 51(1):191–202. doi: 10.1530/JME-13-0021 23674134 PMC3724340

[51] Foord SM Topp SD Abramo M Holbrook JD. New Methods for Researching Accessory Proteins. J Mol Neurosci (2005) 26(2-3):265–76. doi: 10.1385/JMN:26:2-3:265 16012200

[52] Desai AJ Roberts DJ Richards GO Skerry TM. Role of Receptor Activity Modifying Protein 1 in Function of the Calcium Sensing Receptor in the Human TT Thyroid Carcinoma Cell Line. PLoS One (2014) 9(1):e85237. doi: 10.1371/journal.pone.0085237 24454825 PMC3890319

[53] Bouschet T Martin S Henley JM. Receptor-Activity-Modifying Proteins Are Required for Forward Trafficking of the Calcium-Sensing Receptor to the Plasma Membrane. J Cell Sci (2005) 118(Pt 20):4709–20. doi: 10.1242/jcs.02598 PMC3311923 16188935

[54] Mackie DI Nielsen NR Harris M Singh S Davis RB Dy D. RAMP3 Determines Rapid Recycling of Atypical Chemokine Receptor-3 for Guided Angiogenesis. Proc Natl Acad Sci USA (2019) 116(48):24093–9. doi: 10.1073/pnas.1905561116 PMC6883789 31712427

[55] Gao X Zhang D Xu C Li H Caron KM Frenette PS. Nociceptive Nerves Regulate Haematopoietic Stem Cell Mobilization. Nature (2021) 589(7843):591–6. doi: 10.1038/s41586-020-03057-y PMC7856173 33361809

[56] Yoneda T Hiasa M Okui T Hata K. Sensory Nerves: A Driver of the Vicious Cycle in Bone Metastasis? J Bone Oncol (2021) 30:100387. doi: 10.1016/j.jbo.2021.100387 34504741 PMC8411232

[57] Li H Qu J Zhu H Wang J He H Xie X. CGRP Regulates the Age-Related Switch Between Osteoblast and Adipocyte Differentiation. Front Cell Dev Biol (2021) 9:675503. doi: 10.3389/fcell.2021.675503 34124062 PMC8187789

[58] Qi T Dong M Watkins HA Wootten D Miller LJ Hay DL. Receptor Activity-Modifying Protein-Dependent Impairment of Calcitonin Receptor Splice Variant Delta(1-47)hCT((a)) Function. Br J Pharmacol (2013) 168(3):644–57. doi: 10.1111/j.1476-5381.2012.02197.x PMC3579285 22946511

[59] Poyner DR Hay DL. Secretin Family (Class B) G Protein-Coupled Receptors - From Molecular to Clinical Perspectives. Br J Pharmacol (2012) 166(1):1–3. doi: 10.1111/j.1476-5381.2011.01810.x 22489621 PMC3415632

[60] Zirimwabagabo JO Jailani ABA Avgoustou P Tozer MJ Gibson KR Glossop PA. Discovery of a First-In-Class Small Molecule Antagonist Against the Adrenomedullin-2 Receptor: Structure-Activity Relationships and Optimization. J Med Chem (2021) 64(6):3299–319. doi: 10.1021/acs.jmedchem.0c02191 PMC8006142 33666424

[61] Avgoustou P Jailani ABA Zirimwabagabo JO Tozer MJ Gibson KR Glossop PA. Discovery of a First-In-Class Potent Small Molecule Antagonist Against the Adrenomedullin-2 Receptor. ACS Pharmacol Transl Sci (2020) 3(4):706–19. doi: 10.1021/acsptsci.0c00032 PMC7432679 32832872

[62] Garelja ML Walker CS Hay DL. CGRP Receptor Antagonists for Migraine. Are They Also AMY1 Receptor Antagonists? Br J Pharmacol (2021). doi: 10.1111/bph.15585 34076887

[63] Naot D Cornish J. The Role of Peptides and Receptors of the Calcitonin Family in the Regulation of Bone Metabolism. Bone (2008) 43(5):813–8. doi: 10.1016/j.bone.2008.07.003 18687416

[64] Cornish J Callon KE Cooper GJ Reid IR. Amylin Stimulates Osteoblast Proliferation and Increases Mineralized Bone Volume in Adult Mice. Biochem Biophys Res Commun (1995) 207(1):133–9. doi: 10.1006/bbrc.1995.1163 7857256

[65] Cornish J Callon KE King AR Cooper GJ Reid IR. Systemic Administration of Amylin Increases Bone Mass, Linear Growth, and Adiposity in Adult Male Mice. Am J Physiol (1998) 275(4):E694–9. doi: 10.1152/ajpendo.1998.275.4.E694 9755090

[66] Cornish J Callon KE Lin CQ Xiao CL Mulvey TB Coy DH. Dissociation of the Effects of Amylin on Osteoblast Proliferation and Bone Resorption. Am J Physiol (1998) 274(5):E827–33. doi: 10.1152/ajpendo.1998.274.5.E827 9612240

[67] Cornish J Callon KE Lin CQ Xiao CL Gamble GD Cooper GJ. Comparison of the Effects of Calcitonin Gene-Related Peptide and Amylin on Osteoblasts. J Bone Miner Res (1999) 14(8):1302–9. doi: 10.1359/jbmr.1999.14.8.1302 10457262

[68] Rohrs S Kutzner N Vlad A Grunwald T Ziegler S Muller O. Chronological Expression of Wnt Target Genes Ccnd1, Myc, Cdkn1a, Tfrc, Plf1 and Ramp3. Cell Biol Int (2009) 33(4):501–8. doi: 10.1016/j.cellbi.2009.01.016 19353769

[69] Kenny PA Enver T Ashworth A. Receptor and Secreted Targets of Wnt-1/Beta-Catenin Signalling in Mouse Mammary Epithelial Cells. BMC Cancer (2005) 5:3. doi: 10.1186/1471-2407-5-3 15642117 PMC545969

[70] Ziegler S Rohrs S Tickenbrock L Moroy T Klein-Hitpass L Vetter IR. Novel Target Genes of the Wnt Pathway and Statistical Insights Into Wnt Target Promoter Regulation. FEBS J (2005) 272(7):1600–15. doi: 10.1111/j.1742-4658.2005.04581.x 15794748

[71] Wang F Qian H Kong L Wang W Wang X Xu Z. Accelerated Bone Regeneration by Astragaloside IV Through Stimulating the Coupling of Osteogenesis and Angiogenesis. Int J Biol Sci (2021) 17(7):1821–36. doi: 10.7150/ijbs.57681 PMC8120474 33994865

