# Supplementary material for: Accelerated Development With Increased Bone Mass and Skeletal Response to Loading Suggest Receptor Activity Modifying Protein-3 as a Bone Anabolic Target
Source: Front Endocrinol (Lausanne). 2022 Jan 12;12:807882. doi: 10.3389/fendo.2021.807882 (PMC8790142; doi:10.3389/fendo.2021.807882)
Supplement: Supplementary Figure 1 — Protein expression of total β-catenin in differentiating primary osteoblasts. Representative western blot (top) showing increased beta-catenin expression (92kD) in Ramp3 -/- primary osteoblast lysates compared to WTs at day 10,15 and 20 of differentiation. Western blotting was performed for each of the 3 independent osteoblast differentiation experiments. Densitometric analysis (bottom) of the western blots (n=3) confirmed the significance in differential expression of beta-catenin. Level of significance for the difference in gene expression between the genotypes was calculated using the ANOVA test and, is indicated with the number of asterisks (adjusted p value 0.05=*, p value 0.001 = ** so on and so forth). [file DataSheet_1.zip › Supplementary Methods.pdf]

Supplementary Methods document

Dynamic histomorphometry: Dual Calcein labelling

Eight week old mice were studied to determine bone apposition rate by dynamic histomorphometry. Quantification of the bone apposition rate was done using the OsteoMeasure™ system (OsteoMatrics®) on a upright microscope with fluorescence (Leitz DMRB from Leica with Sony DCX-950P 3CCD digital camera).

Specimen processing: Post microCT, left tibiae (fixed in 70% ethanol), were embedded in resin. Resin embedding and sectioning of bone was carried out by the staff members of bone analysis laboratory, core facility (University of Sheffield). Six longitudinal mid sections, 3µm apart, were analysed per specimen for each experiment.

Endocortical dual calcein labels were measures on both lateral and medial sides of the tibia. An off-set of 250µm is left immediately after the growth plate on the lateral side. Six areas of interests 250µm x 250µm totalling 3mm, were then measured on each side of the bone. (Fig. 2.7).

Calculations: Bone apposition rate was calculated using the following formula:

Bone apposition rate= Inter label thickness (In.L.Th) / time between labels (day).

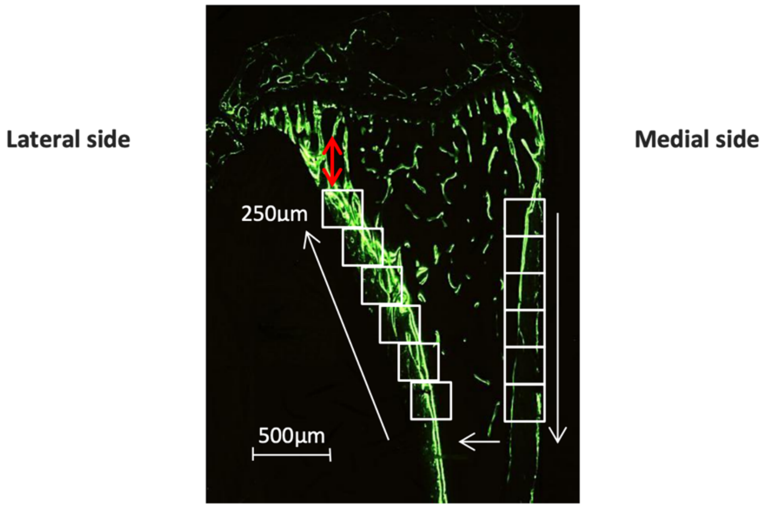

Figure 1: Representative image depicting the method used to measure the endocortical dual Calcein labels in Tibia. An offset of 250µm (red double headed arrow) is left after the growth plate. The first 500µm field of interest starts after the offset. In total, 6 x 500µm areas (3mm) were measured on each, lateral and medial side. Arrows mark the direct of measurement (successive field of interests).

Haematoxylin and Eosin (H&E) staining and Tartrate-resistant acid phosphatase (TRAP) staining

Specimen processing: Right tibiae were fixed in ice cold 4%paraformaldehyde. Bones were then decalcified and embedded in paraffin wax blocks before sectioning and staining (bone analysis laboratory, core facility - University of Sheffield). Briefly bones were decalcified in EDTA at room temperature for 4 weeks using 10-20 times volume of EDTA to volume of bone. The EDTA solution was changed each week. On completion of decalcification process, bones were embedded in paraffin wax and sectioned. Out of the six longitudinal mid sections 3µm apart, three alternate sections were used for H&E (1) staining and the other three sections were used for TRAP(2) staining (bone analysis laboratory, core facility - University of Sheffield).
H&E stained sections were used to study the bone versus cartilage differences. These sections were also used to determine the differences between the ratio of proliferative to hypertrophic zones in the growth plates. TRAP stained sections were used to determine the number of osteoblast and osteoclasts and the osteoblast-osteoclast coverage. These measurements were performed on both the endocortical and the trabecular surface of tibia.
Virtual sections: H&E and TRAP sections were scanned in the ScanScope® (Aperio®) scanner. All histological analyses were then carried out manually on virtual sections in ImageScope™ (Aperio®), the e-slide viewing software.

Virtual analysis

i. Trabecular area
Scanned images of the H&E and TRAP sections were uploaded in the ImageScope™ software. Trabecular area in the section was measured by marking a ~2.0mm distance in the marrow cavity from the midpoint of the growth-plate. The trabecular region in the section of the bone was manually marked in an overlapping virtual layer. The software then simultaneously calculates the number of trabecular areas manually drawn and the area of each trabecular unit (Figure 2).

The average thickness of the trabecular units was calculated with the formula:

Trabecular thickness: Total area of all the trabecular units / Number of trabecular units

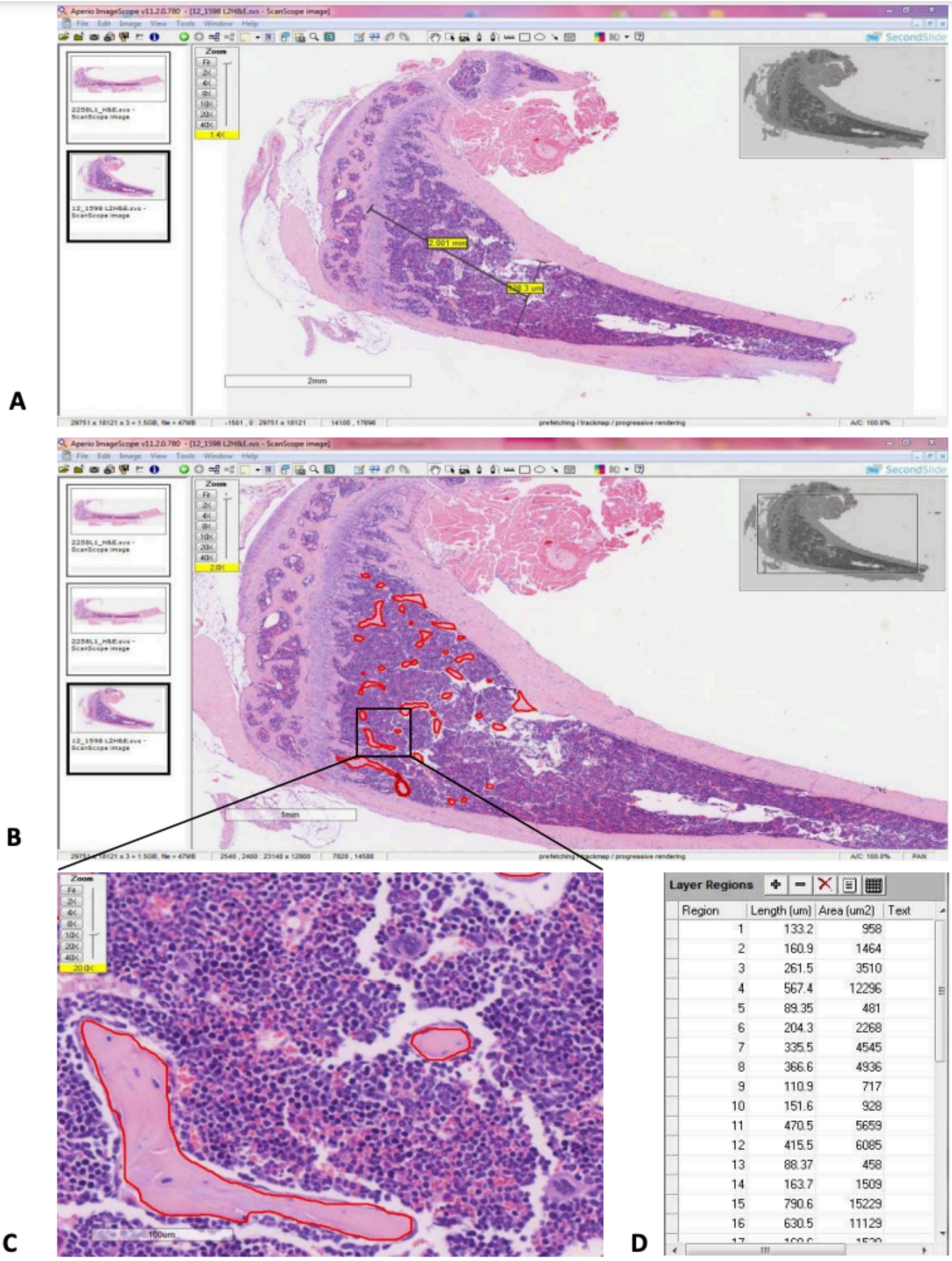

Figure 2: Representative image depicting the method used to quantify the trabecular area in e-slide using ImageScope®. The top panel is a screen-capture showing the marking of 2.0mm distance from the growth (A). Middle panel (B) shows the region of interests drawn around the trabecular units. Panel C, is a zoomed image showing the accuracy of trabecular marking. The measurements of marked regions generated by the software is shown in panel D.

ii. Osteoblast and osteoclast numbers:
Number of Osteoclasts (N.Ob.), osteoblast covered bone surface (Ob.S.), number of osteoclasts (N.Oc.) and osteoclast covered bone surface (Oc.S.) were determined on both the endocortical surface of the bone and the trabecular bone. Cells were manually marked on the viewing software. Active osteoblasts were quantified. They were differentiated from the bone lining cells by their cuboidal shape. Osteoclasts with 3 or more nuclei were quantified. Similar to the dual calcein labels, the endocortical measurements were done in 12, 300μm x 300μm regions: 6 on the medial and 6 on the lateral side. Trabecular measurements were done in a 750μm x 750μm area. An offset of 300μm from the growth plate was maintained in both endocortical and trabecular measurement (Figure 3).

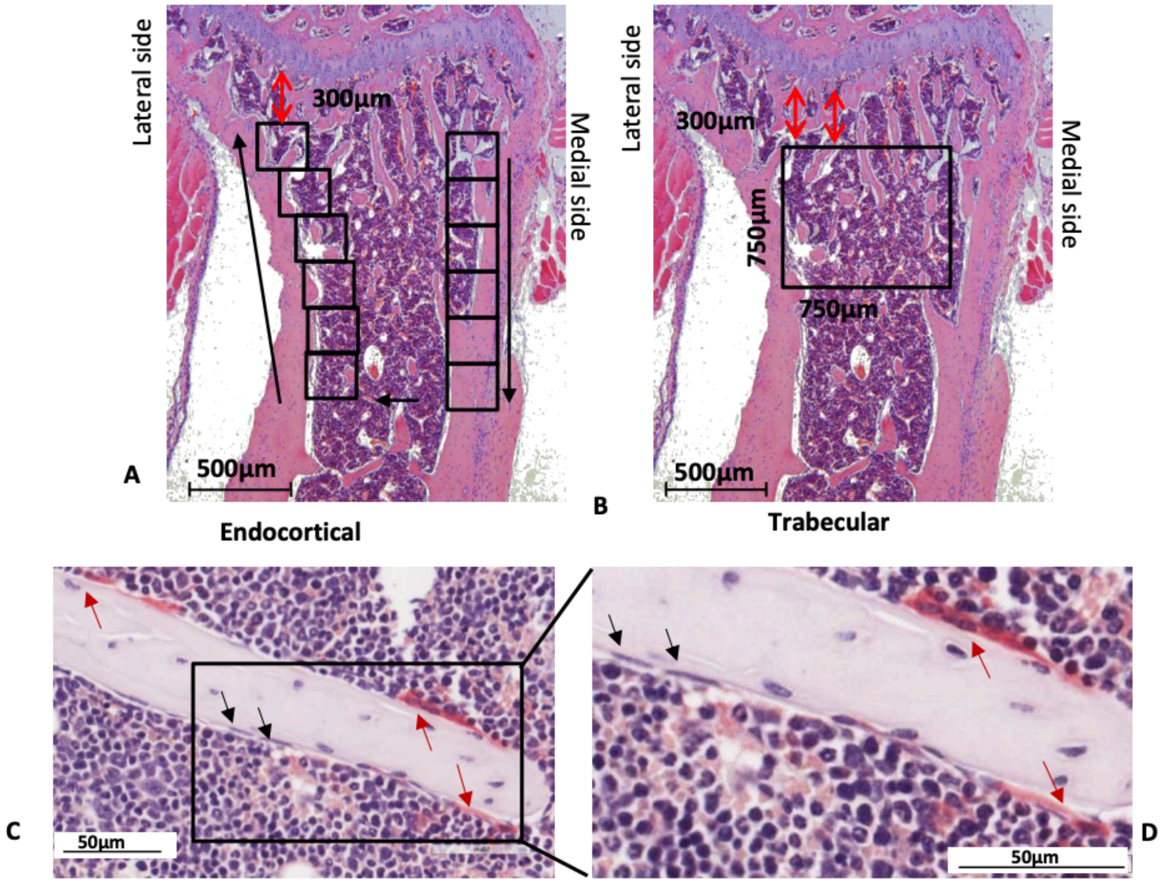

Figure 3: Panels A and B representative images depicting the method used to measure the endocortical (A) and trabecular (B) bone cell measurements in tibia. An offset of 300μm (red double headed arrow) is left after the growth plate. Panels C and D (zoomed ROI of C) show TRAP positive osteoclast in red (Red arrows) and osteoblasts lined on the bone surface (black arrows).

iii. Growth plate analysis:
The proliferative and hypertrophic zones of the growth plate were studied in order to characterise the growth plate mice. Using virtual slides, number of proliferative cells (N.PC), number of hypertrophic cells (N. HC) and the ratio of proliferative zone to hypertrophic zone (PC:HC) were determined by manually marking the cells. Three H&E stained tibial longitudinal mid sections (6µm apart) were analysed per sample. An area of 1mm x 0.6mm mid growth plate region was analysed in each section. First, individual chondrocyte columns were manually marked as each column represents a clonal expansion of stem cells (3). Then proliferative and hypertrophic chondrocytes were marked manually with different colours in overlapping layers. Finally the extent of proliferative zone and hypertrophic zone within each column was marked. The software then simultaneously calculated the number of cells and PC/HC zone. Figure 4 below, details this process.

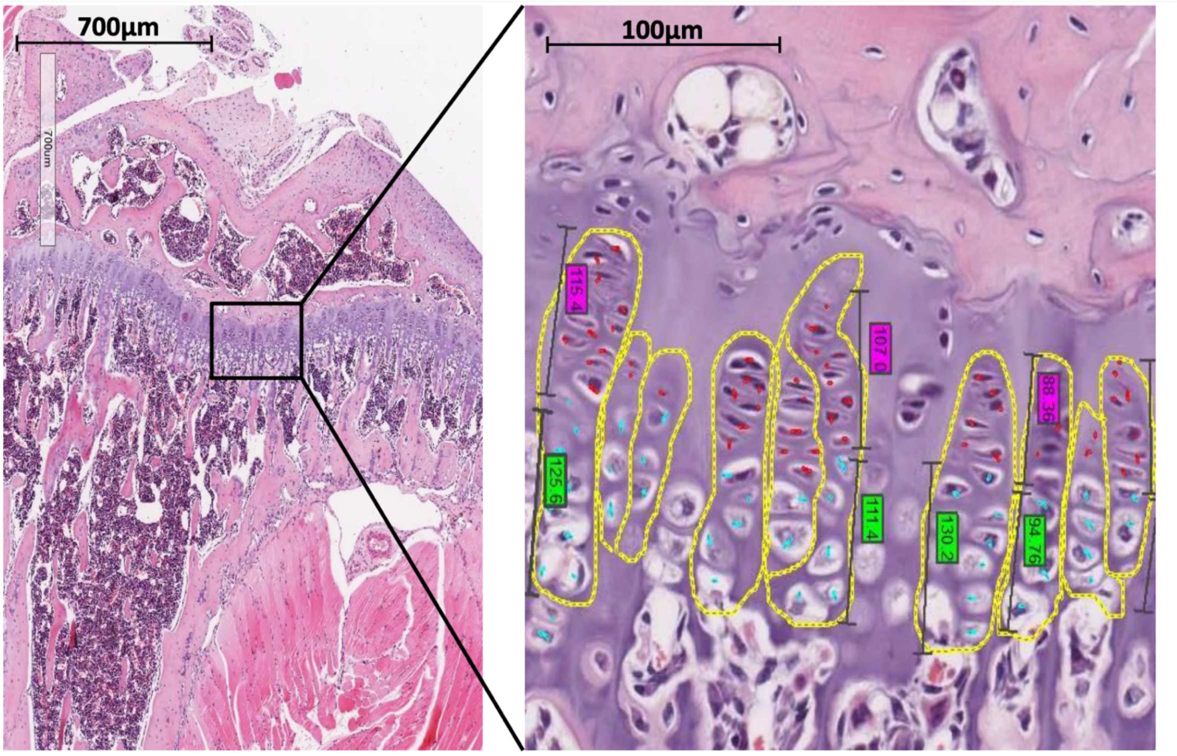

Figure 4: Representative image depicting the method used to analyse growth plate. The left panel is a tibial section with the manually marked analysis region (black box). The right panel is the zoomed region of analysis with the different markings. The yellow manual drawing marks individual chondrocyte column, pink dots mark proliferative cells, light blue dots mark the hypertrophic cells. Proliferative zone length is marked by black line with pink label, whereas the hypertrophic zone length is marked with black line with green label.

References

1. Cardiff RD, Miller CH, Munn RJ. Manual hematoxylin and eosin staining of mouse tissue sections. Cold Spring Harb Protoc. 2014;2014(6):655-8.
2. Yang J, Bi X, Li M. Osteoclast Differentiation Assay. Methods Mol Biol. 2019;1882:143-8.
3. Farnum CE, Wilsman NJ. Determination of proliferative characteristics of growth plate chondrocytes by labeling with bromodeoxyuridine. Calcif Tissue Int. 1993;52(2):110-9.
