# Supplementary material for: Accelerated Development With Increased Bone Mass and Skeletal Response to Loading Suggest Receptor Activity Modifying Protein-3 as a Bone Anabolic Target
Source: Front Endocrinol (Lausanne). 2022 Jan 12;12:807882. doi: 10.3389/fendo.2021.807882 (PMC8790142; doi:10.3389/fendo.2021.807882)
Supplement: Supplementary Figure 1 — Protein expression of total β-catenin in differentiating primary osteoblasts. Representative western blot (top) showing increased beta-catenin expression (92kD) in Ramp3 -/- primary osteoblast lysates compared to WTs at day 10,15 and 20 of differentiation. Western blotting was performed for each of the 3 independent osteoblast differentiation experiments. Densitometric analysis (bottom) of the western blots (n=3) confirmed the significance in differential expression of beta-catenin. Level of significance for the difference in gene expression between the genotypes was calculated using the ANOVA test and, is indicated with the number of asterisks (adjusted p value 0.05=*, p value 0.001 = ** so on and so forth). [file DataSheet_1.zip › Supplementary Table 1.pdf]

Bone morphometric parameter	Units	WT Mean ± SEM.	Ramp3 KO Mean ± SEM.	P Value	Significance
**PND5 male femur BV**	mm^3	0.33 ± 0.029 n=9	0.47 ± 0.047 n=5	0.0189	*
**PND5 female femur BV**	mm^3	0.31 ± 0.02 n=4	0.56 ± 0.70 n=9	0.0435	*
**4 week female whole femur BV**	mm^3	2.90 ± 0.11 n=8	3.33 ± 0.12 n=9	0.0201	*
**4 week female whole tibia-fibula BV**	mm^3	3.95 ± 0.16 n=8	4.64 ± 0.11 n=9	0.0022	**
**4 week female femur cortical BV**	mm^3	0.45 ± 0.00 n=10	0.45 ± 0.00 n=9	0.7526	NS
**4 week female femur cortical Th**	mm	0.10 ± 0.00 n=10	0.10 ± 0.00 n=11	0.9901	NS
**4 week female tibia cortical BV**	mm	0.51 ± 0.00 n=9	0.51 ± 0.01 n=10	0.7961	NS
**4 week female tibia cortical Th**	mm	0.08 ± 0.00 n=9	0.09 ± 0.00 n=11	0.8290	NS
**4 week female femur trabecular BV**	mm^3	0.18 ± 0.01 n=9	0.17 ± 0.011 n=9	0.7137	NS
**4 week female femur trabecular BV/TV**	%	5.22 ± 0.17 n=9	5.00 ± 0.20 n=11	0.4169	NS
**4 week female femur trabecular Th**	mm	0.03 ± 0.00 n=9	0.035 ± 0.00 n=9	0.002	***
**4 week female femur trabecular Sp**	mm	0.29 ± 0.01 n=9	0.32 ± 0.01 n=11	0.1298	NS
**4 week female femur trabecular N**	1/mm	1.65 ± 0.06 n=9	1.47 ± 0.06 n=11	0.0477	*
**4 week female femur trabecular Pf**	1/mm	27.87 ± 1.77 n=9	32.22 ± 0.97 n=9	0.0471	*
**4 week female tibia trabecular BV**	mm^3	0.10 ± 0.01 n=8	0.10 ± 0.01 n=10	0.7631	NS
**4 week female tibia trabecular BV/TV**	%	3.19 ± 0.14 n=8	3.19 ± 0.19 n=10	0.9916	NS
**4 week female tibia trabecular Th**	mm	0.03 ± 0.00 n=7	0.04 ± 0.00 n=10	0.0024	**
**4 week female tibia trabecular Sp**	mm	0.43 ± 0.02 n=8	0.44 ± 0.03 n=10	0.6506	NS
**4 week female tibia trabecular N**	1/mm	0.94 ± 0.05 n=8	0.92 ± 0.05 n=10	0.7370	NS
**4 week female tibia trabecular Pf**	1/mm	36.80 ± 0.76 n=7	38.15 ± 0.89 n=10	0.2970	NS
**4 week female caudal vertebra BV**	mm^3	1.24 ± 0.08 n=8	1.48 ± 0.07 n=8	0.0324	*
**4 week female caudal vertebra Th**	mm	0.06 ± 0.00 n=8	0.07 ± 0.00 n=8	0.0806	NS
