# Supplementary material for: Accelerated Development With Increased Bone Mass and Skeletal Response to Loading Suggest Receptor Activity Modifying Protein-3 as a Bone Anabolic Target
Source: Front Endocrinol (Lausanne). 2022 Jan 12;12:807882. doi: 10.3389/fendo.2021.807882 (PMC8790142; doi:10.3389/fendo.2021.807882)
Supplement: Supplementary Figure 1 — Protein expression of total β-catenin in differentiating primary osteoblasts. Representative western blot (top) showing increased beta-catenin expression (92kD) in Ramp3 -/- primary osteoblast lysates compared to WTs at day 10,15 and 20 of differentiation. Western blotting was performed for each of the 3 independent osteoblast differentiation experiments. Densitometric analysis (bottom) of the western blots (n=3) confirmed the significance in differential expression of beta-catenin. Level of significance for the difference in gene expression between the genotypes was calculated using the ANOVA test and, is indicated with the number of asterisks (adjusted p value 0.05=*, p value 0.001 = ** so on and so forth). [file DataSheet_1.zip › Supplementary Table 2.pdf]

Bone morphometric parameter	Units	WT Mean ± SEM.	Ramp3 KO Mean ± SEM.	P Value	Significance
**8 week female whole femur BV**	mm^3	10.28 ± 0.45 n=4	10.69 ± 0.24 n=4	0.4594	NS
**8 week female whole tibia-fibula BV**	mm^3	11.03 ± 0.28 n=4	11.17 ± 0.18 n=4	0.6704	NS
**8 week female femur cortical BV**	mm^3	0.71 ± 0.02 n=4	0.70 ± 0.02 n=4	0.8408	NS
**8 week female femur cortical Th**	mm	0.21 ± 0 n=4	0.2 ± 0.01 n=4	0.4263	NS
**8 week female tibia cortical BV**	mm^3	0.74 ± 0.02 n=4	0.84 ± 0.07 n=4	0.2588	NS
**8 week female tibia cortical Th**	mm	0.18 ± 0.06 n=4	0.18 ± 0 n=4	0.6396	NS
**8 week female femur trabecular BV**	mm^3	0.3 ± 0.01 n=4	0.31 ± 0.01 n=4	0.6110	NS
**8 week female femur trabecular BV/TV**	%	8.0 ± 0.3 n=4	8.6 ± 0.3 n=4	0.1899	NS
**8 week female femur trabecular Th**	mm	0.04 ± 0.001 n=4	0.05 ± 0.0012 n=4	0.0299	*
**8 week female femur trabecular Sp**	mm	0.32 ± 0.011 n=4	0.32 ± 0.003 n=4	0.9321	NS
**8 week female femur trabecular N**	1/mm	1.89 ± 0.05 n=4	1.91 ± 0.08 n=4	0.8665	NS
**8 week female femur trabecular Pf**	1/mm	20.6 ± 1.0 n=4	19.3 ± 0.5 n=4	0.2913	NS
**8 week female tibia trabecular BV**	mm^3	0.13 ± 0.01 n=4	0.13 ± 0.01 n=4	0.7735	NS
**8 week female tibia trabecular BV/TV**	%	4. ± 0.2 n=4	5.25 ± 0.22 n=4	0.0058	**
**8 week female tibia trabecular Th**	mm	0.04 ± 0.001 n=4	0.05 ± 0.001 n=4	0.0436	*
**8 week female tibia trabecular Sp**	mm	0.53 ± 0.01 n=4	0.42 ± 0.03 n=4	0.0150	*
**8 week female tibia trabecular N**	1/mm	0.93 ± 0.04 n=4	1.17 ± 0.04 n=4	0.0047	**
**8 week female tibia trabecular Pf**	1/mm	29.23 ± 2.5 n=4	28.43 ± 0.74 n=4	0.7696	NS

Bone morphometric parameter	Units	WT Mean ± SEM.	Ramp3 KO Mean ± SEM.	P Value	Significance
**8 week male inter Calcein label width**	μm	10.66 ± 0.85 n=5	14.88 ± 1.19 n=6	0.0218	*
**8 week male bone apposition rate**	μm/day	1.52 ± 0.12 n=5	2.12 ± 0.17 n=6	0.0223	*
**8 week male tibia total trabecular area**	μm^2	187746 ± 14532 n=5	265877 ± 20627 n=5	0.0147	*
**8 week male tibia total trabecular surface**	μm	13422 ± 959.4 n=5	16074 ± 1327 n=5	0.1440	NS
**8 week male tibia average trabecular area per trabecular unit (trabecular thickness)**	μm^2	5566 ± 525.5 n=5	8365 ± 1077 n=5	0.0477	*
**8 week male tibia total number of trabecular units**	Arbitrary number	34.00 ± 1.30 n=5	33.20 ± 3.43 n=5	0.8327	NS
