# Supplementary figures and images for: Accelerated Development With Increased Bone Mass and Skeletal Response to Loading Suggest Receptor Activity Modifying Protein-3 as a Bone Anabolic Target
Source: Front Endocrinol (Lausanne). 2022 Jan 12;12:807882. doi: 10.3389/fendo.2021.807882 (PMC8790142; doi:10.3389/fendo.2021.807882)

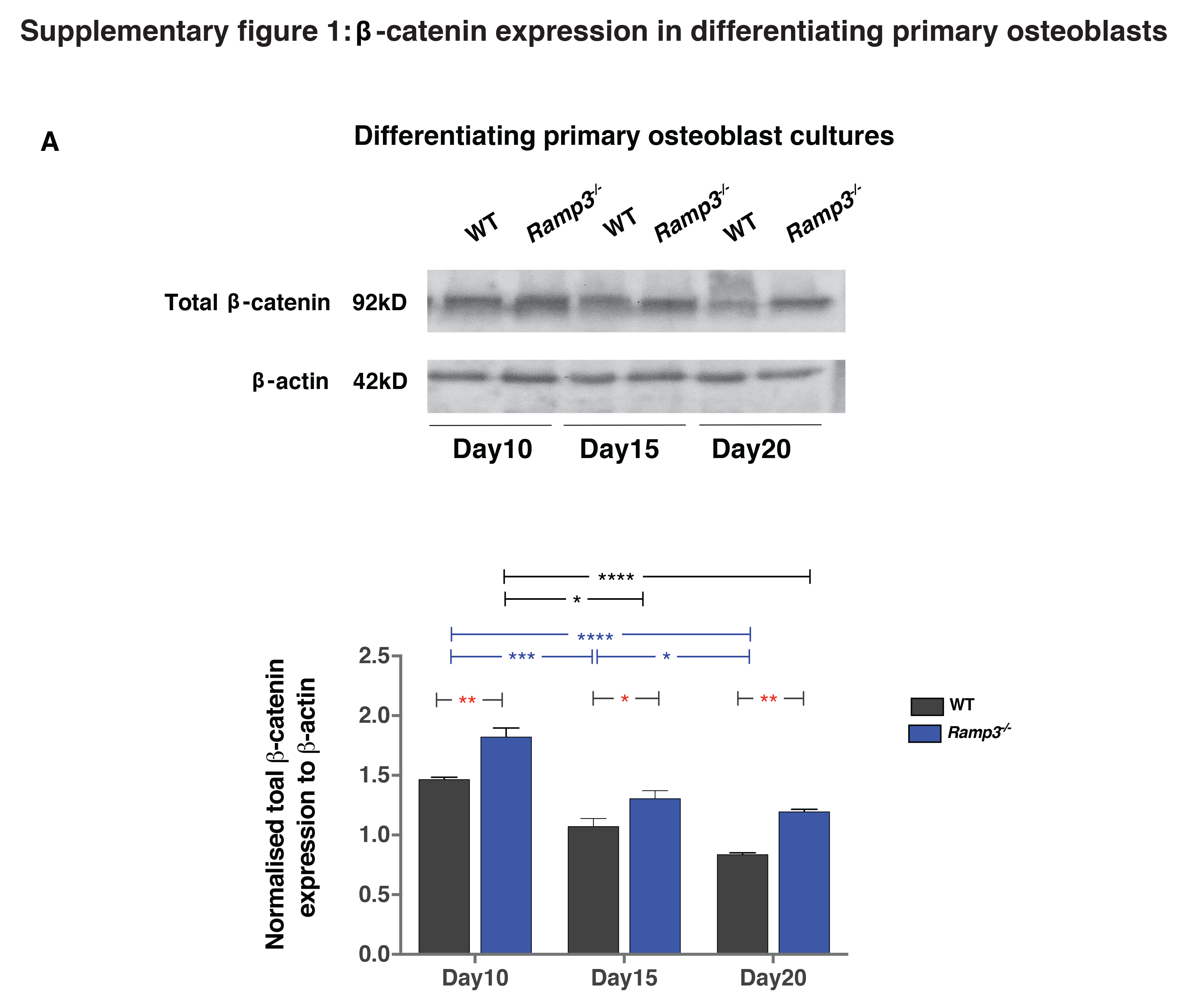

Supplement: Supplementary Figure 1 — Protein expression of total β-catenin in differentiating primary osteoblasts. Representative western blot (top) showing increased beta-catenin expression (92kD) in Ramp3 -/- primary osteoblast lysates compared to WTs at day 10,15 and 20 of differentiation. Western blotting was performed for each of the 3 independent osteoblast differentiation experiments. Densitometric analysis (bottom) of the western blots (n=3) confirmed the significance in differential expression of beta-catenin. Level of significance for the difference in gene expression between the genotypes was calculated using the ANOVA test and, is indicated with the number of asterisks (adjusted p value 0.05=*, p value 0.001 = ** so on and so forth). [file DataSheet_1.zip › Supplementary Figure 1.TIF]
